# Fear of Being Ordinary: Development and Validation of a Questionnaire

**DOI:** 10.3390/ejihpe16100166

**Published:** 2026-10-19

**Authors:** Anne Schienle, Arved Seibel

**Affiliations:** Clinical Psychology, University of Graz, 8010 Graz, Austria

**Keywords:** fear of being ordinary (FoBO), ordinary life, fear of missing out (FoMO), social media, Big Five, narcissism, social comparison, scale development, emotional distress, online survey

## Abstract

Social media often presents staged portrayals of individuals’ lives, creating the impression that others have exciting and extraordinary experiences. This phenomenon can contribute to unrealistic social comparisons and concerns about living an ordinary life. The present study sought to develop a psychometrically robust measure of individual differences in fear of being ordinary (FoBO) using online survey data from a convenience sample of 622 participants (M = 21.88 years; 69% female). Furthermore, the study explored associations between FoBO, fear of missing out (FoMO), the Big Five personality dimensions, narcissistic traits, and levels of self-reported psychological distress. Exploratory and confirmatory factor analyses revealed two correlated factors in the FoBO questionnaire: fear of insignificance (6 items) and social media comparisons (3 items). FoBO showed the strongest positive correlations with FoMO (r = 0.58), depression (r = 0.40), and neuroticism (r = 0.34). Daily time spent on social media was also positively associated with FoBO (r = 0.26). In conclusion, the brief FoBO questionnaire provides a useful tool for further research on the relationship between intense social media use and this specific fear, as well as for evaluating potential strategies to mitigate it.

## 1. Introduction

Social media use is very popular, particularly among young people. However, frequent engagement has been linked to specific concerns and fears, as well as lower levels of life satisfaction (Orben et al., 2022). One of these concerns has been labeled ‘fear of missing out’ (FoMO), describing apprehensions that one is not in the know or missing out on information, events, experiences, or life decisions that could make one’s life more fulfilling (Przybylski et al., 2013; Tandon et al., 2021). More specifically, FoMO refers to the anxiety and worries resulting from not participating in meaningful and pleasurable activities in which others are currently involved (Abel et al., 2016). Przybylski et al. (2013) propose that FoMO is a negative emotional state resulting from unmet social relatedness needs.

Elevated FoMO has been linked to adverse health outcomes, including depressive symptoms, increased anxiety and poorer sleep quality (Gupta & Sharma, 2021). In addition, FoMO tends to co-occur with specific personality characteristics, showing positive relationships with narcissism and neuroticism and inverse relationships with conscientiousness, agreeableness, and openness (Rozgonjuk et al., 2021; Servidio et al., 2021). Supporting these findings, a meta-analysis of 35 studies (n = 18,964) reported that individuals with higher FoMO levels exhibited greater neuroticism and lower conscientiousness (Zhang et al., 2024).

A recent social media phenomenon that might be related to FoMO has been termed koinophobia (Koenig, 2021). Koinophobia (from ancient Greek koinós: common, ordinary, stripped of specialness and phobos: fear) refers to a condition where an individual has an (irrational) fear of leading an ordinary or unremarkable life, often coupled with an anxiety about having an unfulfilled existence or failing to achieve extraordinary things. To the best of our knowledge, koinophobia, or the “fear of being ordinary” (FoBO), has not yet been investigated empirically, although the construct has been discussed in theoretical writings. For example, Kelly (2011) conceptualized adolescence as a developmental period during which a fear, or even terror, of being ordinary may emerge and motivate behaviors aimed at making one’s life less ordinary. Optimal Distinctiveness Theory (Brewer, 1991) proposes that individuals strive to balance two fundamental identity motives: the need for belonging and inclusion within social groups and the need to maintain a sense of distinctiveness from others (Leonardelli et al., 2010). From this perspective, threats to the need for belonging may contribute to FoMO, whereas insufficient fulfillment of the need for distinctiveness may contribute to FoBO. These concerns may be particularly salient in the context of social media, where curated and idealized portrayals of others’ lives provide frequent opportunities for social comparison and may amplify perceptions of being insufficiently connected, or distinctive.

From a conceptual standpoint, it is also important to establish that FoBO can be differentiated from related constructs that have already been introduced in the literature. For example, individuals high in narcissistic traits may experience a strong need to feel special, unique, and non-ordinary. In particular, narcissistic grandiosity is characterized by an exaggerated sense of self-importance, superiority, and uniqueness (see the Narcissism Spectrum Model; Krizan & Herlache, 2018).

The purpose of the present study was therefore to develop and validate a questionnaire for the assessment of FoBO. For the validation, scores on the new FoBO scale were correlated with the FoMO scale (Przybylski et al., 2013) and questionnaires for the assessment of the Big Five personality traits (Rammstedt et al., 2013), narcissism (von Collani, 2008), and reported symptoms of depression, anxiety, and somatization (Spitzer et al., 2011). We formulated the following hypotheses regarding the validity (convergent, discriminant) and reliability of the new FoBO scale. We expected FoBO to be moderately positively associated with constructs reflecting concerns about social disconnection, including social media use and FoMO (Przybylski et al., 2013), as well as with a desire to feel special, reflected in narcissistic tendencies (von Collani, 2008). Given previous findings linking FoMO to indicators of poorer psychological well-being, such as neuroticism, depression, and anxiety (e.g., Gupta & Sharma, 2021; Zhang et al., 2024), we expected FoBO to show similar associations with these indicators. We also expected that FoBO will show weak associations with theoretically and empirically unrelated personality dimensions, such as extraversion, supporting the discriminant validity of the FoBO scale. Finally, assuming that FoBO has relatively stable, trait-like characteristics, we expected the scale to demonstrate high test–retest reliability.

## 2. Materials and Methods

### 2.1. Participants

The convenience sample consisted of 622 participants (M = 21.88 years, SD = 7.71; age range: 14–65 years; 69% female). Inclusion criteria were being at least 14 years of age and fluent in German.

### 2.2. Measures

Participants completed the following questionnaires to assess the construct validity of the new scale.

The **Big-Five-Inventory-10** (BFI-10; German version by Rammstedt et al., 2013) is a 10-item questionnaire developed to measure the personality traits: openness to experience (e.g., “I see myself as someone who has an active imagination.”), conscientiousness (e.g., “I see myself as someone who does a thorough job.”), extraversion (“I see myself as someone who is outgoing and sociable.”), agreeableness (e.g., “I see myself as someone who is generally trusting.”), and neuroticism (e.g., “I see myself as someone who gets nervous easily.”). Each item is rated on a 5-point Likert scale (1 = strongly disagree, 5 = strongly agree). The scale was developed based on the 44-item Big Five Inventory (BFI-44; John et al., 1991). This is a well-established measure of the five major personality dimensions and has demonstrated cross-cultural applicability.

The **Fear of Missing Out Scale** (Original version by Przybylski et al., 2013; German version by Rozgonjuk et al., 2020) consists of 10 items designed to assess the extent of an individual’s fear of missing out (FoMO), especially on positive social experiences. Items (e.g., “I get worried when I find out my friends are having fun without me.”) are rated on a 5-point Likert scale (1 = not at all true of me; 5 = extremely true of me). Total scores are calculated by averaging responses, with higher scores indicating higher levels of FoMO.

The short version of the **Narcissistic Personality Inventory** (NPI-10, German version by von Collani, 2008) aims to capture narcissistic traits (e.g., “It is easy for me to manipulate others.”). Responses are given on 5-point scales (1 = does not apply at all; 5 = fully applies). A higher score indicates a more pronounced narcissistic personality. The NPI-10 does not provide information about the presence of clinically relevant symptoms of narcissistic personality disorder. The original NPI was developed by Raskin and Terry (1988).

The short version of the **Brief Symptom Inventory** (BSI-18; German version by Spitzer et al., 2011) is an easily applicable, reliable and valid self-report measure in wide international use. It assesses the symptom dimensions of somatization (e.g., nausea, difficulties breathing), depression (e.g., lack of interest, loneliness) and anxiety (e.g., nervousness, tension). The respondents rate how much they have been distressed by each of the 18 symptoms during the last week using 5-point scales (0 = not at all; 4 = extremely). Higher scores reflect a higher degree of psychological distress. The original BSI was developed by Derogatis (1993).

### 2.3. Scale Development

#### 2.3.1. Domain Identification and Item Generation

The construct under investigation, fear of being ordinary (FoBO), was defined as “distress or anxiety in the face of the perceived ordinariness of one’s own life” (Koenig, 2021). A review of the existing literature indicated that no established instruments were available for assessing FoBO.

As a starting point for item generation, we conducted an exploratory online search, drawing from blog posts and forum content for inspiration. This was done so the items would capture the lived experiences of the phenomenon by the target population. We refrained from using reverse-coded items to avoid potential distortions in factor loadings. The initial item pool (40 items) was evaluated by experts (four clinical psychologists) and four students as part of a content validity analysis. This led to a reduction in the item pool to 15 items. In a second round of evaluation, four additional items were removed because they were found to not fully align with the construct. Items were supposed to reflect dissatisfaction with leading a life that is perceived as having no lasting impact as well as negative emotions evoked through comparisons of one’s own life to that of others, particularly on social media. It was attempted to create a measure with a similar number of items as the FoMO scale. The resulting 11 items are shown in Table 1.

We used a five-point Likert-type response scale with the endpoints labeled (1 = does not apply at all; 5 = applies completely), as recommended for unipolar items (see Boateng et al., 2018, for best-practice guidelines on developing and validating scales for health, social, and behavioral research).

#### 2.3.2. Survey Administration and Sample Size

Regarding sample size, we followed the recommendations by Clark and Watson (1995) to include at least 300 respondents in the survey. The survey was administered to a convenience sample that included younger participants with social media use, reflecting the target population. To assess the test–retest reliability of the new FoBO scale, participants of a second survey completed the scale twice, with a one-week interval between assessments.

### 2.4. Procedure

Survey 1: We conducted a fully anonymized open online survey that was advertised via social media and mass email distributions to students at the local university. The survey asked for demographic data (age, gender), social media use (duration, platforms) and included several questionnaires (see Section 2.2). The survey was accessed 610 times, and 492 participants that fit the inclusion criteria completed it. One participant was excluded due to implausible extreme responses (reported social media use of 809 h per day) and straightlining response behavior resulting in a final sample of 491 participants (M = 21.93 years, SD = 8.46; 67% female). Three participants reported daily social media use of 20 h or more, which was considered implausible; therefore, this variable was set to missing for these cases.

Survey 2: Data from a second online survey (n = 131; M = 21.69 years, SD = 3.75; 79% female) were collected to examine the temporal stability of FoBO. Of these participants, 120 completed the FoBO questionnaire at both measurement points.

All participants were informed in advance about the structure (number of questions) and the objectives of the study as well as the ethical aspects of the study, their rights as participants, and data protection and confidentiality procedures; their informed consent was obtained. Participants received no financial compensation.

Non-response options for the questionnaires were not included in the survey. Consistency and completeness checks were conducted after the survey had been completed. No technical mechanism was used to prevent repeated participation by the same individual (e.g., IP addresses were not collected). Only data from participants who completed the survey were included in the analyses. Reporting followed the guidelines provided by Turk et al. (2018).

### 2.5. Statistical Analysis

#### 2.5.1. Scale Evaluation

To examine the factor structure of the FoBO questionnaire, we conducted an exploratory factor analysis (EFA) on a randomly selected subsample comprising half of the available data (n = 311; M = 22.10 years; 64% female). Specifically, we used principal axis factoring with oblimin rotation as we assumed potential factors to be correlated (Fabrigar et al., 1999). We used parallel analysis to determine the number of factors to retain (Horn, 1965).

Subsequently, we tested the fit of the identified factor structure with confirmatory factor analysis (CFA) using the data of the remaining 311 participants (M = 21.66 years, 75% female). No missing data were present in the CFA sample. The CFA was conducted using the maximum likelihood estimation method. We used the chi-square test (χ^2^), comparative fit index (CFI), Tucker–Lewis index (TLI), root mean square error of approximation (RMSEA), and standardized root mean square residual (SRMR) to evaluate model fit (Brown, 2015; Nye, 2023). CFI and TLI values close to or greater than 0.95, RMSEA values close to or below 0.06 and SRMR values close to or below 0.08 were considered indicative of good model fit (Hu & Bentler, 1999).

#### 2.5.2. Construct Validity and Retest-Reliability

We report zero-order correlations of FoBO with FoMO, narcissism, daily time spent on social media, reported symptoms of depression, anxiety, somatization, the Big Five personality traits (openness, conscientiousness, extraversion, agreeableness, and neuroticism) and age as part of construct validation.

Moreover, test–retest reliability was assessed for a one-week interval. Independent-sample *t*-tests were conducted to explore gender differences in FoBO and time spent on social media. Data analysis was conducted using Jamovi 2.6.44.0 and IBM SPSS Statistics version 29.0.0.0.

## 3. Results

### 3.1. Factor Analyses

An overall KMO value of 0.86 and the Bartlett’s test of sphericity (χ^2^(36) = 1284.36, *p* < 0.001) indicated that EFA was suitable for the data. We iteratively eliminated items with low primary loading, high cross-loadings and/or high uniqueness. The most problematic item was removed first and the analysis was subsequently repeated. This resulted in a final set of nine items: six items loading on factor 1 and three items on factor 2. Thematically, factor 1 refers to worries and fears about falling short on leaving an impact on the world as well as leading a life that goes unrecognized by others (Fear of Insignificance). Factor 2 is centered around negative emotionality through comparisons on social media (Social Media Comparisons). McDonald’s omega in the EFA sample was 0.86 for the Fear of Insignificance subscale and 0.85 for the Social Media Comparisons subscale.

**Figure 1:**
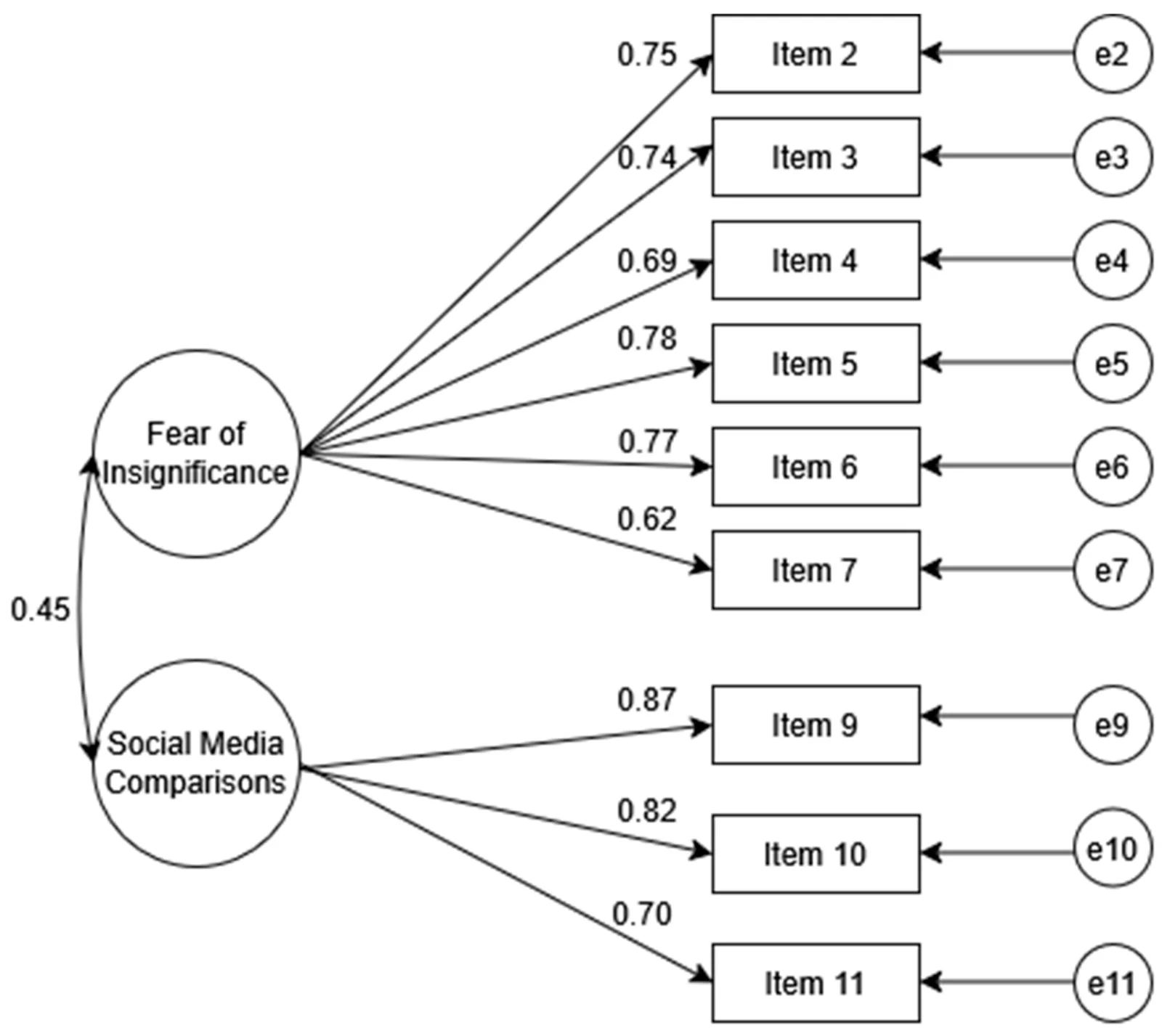
Path diagram with two-factor structure. Note. All estimates are standardized using the Jamovi module semlj (version 1.2.5).

The CFA supported the two-factor solution. Although the test for the exact model fit was significant, χ^2^(26) = 49.75, *p* = 0.003, all other indices indicated a good fit of the model (CFI = 0.98; TLI = 0.97; RMSEA = 0.05, 90% CI [0.03, 0.08]; SRMR = 0.03). Standardized factor loadings ranged from 0.62 to 0.87 and were all statistically significant (Figure 1). The two factors were moderately correlated, r = 0.45, *p* < 0.001, based on the CFA (For more detailed information on the item-level descriptives in the CFA, see Supplementary Table S1).

Additionally, to evaluate whether calculating a total scale score would be appropriate, we conducted a CFA testing a one-factor model with correlated error terms for the three items related to social media comparisons. This model also showed a good fit to the data (χ^2^(24) = 49.18, *p* = 0.002; CFI = 0.98; TLI = 0.97; RMSEA = 0.06, 90% CI [0.03, 0.08]; SRMR = 0.03). All standardized factor loadings were significant, ranging from 0.33 to 0.78. Given the good model fit and the reasonable assumption of an underlying factor, the calculation of an overall average score appears to be justified. Based on the simulation results reported by Wolf et al. (2013), the sample size can be considered adequate for both of the present CFA models.

### 3.2. Descriptive Statistics, Bivariate Correlations and Gender Comparisons

The total scores of the FoBO questionnaire were significantly associated with all measured variables, except for extraversion and openness to experience (see Table 2). For additional descriptive statistics and detailed information on the correlations, see Supplementary Tables S2 and S3.

The highest positive correlations emerged with FoMO. We found moderate positive associations with psychological distress (reported symptoms of depression, anxiety, and somatization), neuroticism, narcissistic traits, and daily time spent on social media. Agreeableness and conscientiousness were negatively correlated with all FoBO scores (total and subscale scores). Moreover, younger participants tended to report higher levels of FoBO. The test–retest reliabilities for the subscales and for the total scale were high (Fear of Insignificance: r = 0.80; Social Media Comparisons: r = 0.81; Total Scale: r = 0.81).

Females (M = 2.35, SD = 0.88) reported higher levels of FoBO (total score) than males (M = 2.18, SD = 0.86; t(607) = 2.19, *p* = 0.029, d = 0.20). This difference was driven by the subscale Social Media Comparisons with significantly higher scores in the female group (M = 2.46, SD = 1.11) than in the male group (M = 1.93, SD = 1.00; t(364.35) = 5.75, *p* < 0.001, d = 0.50). Scores on the subscale Fear of Insignificance did not differ between genders (*p* = 0.91, d = 0.01).

Participants reported the use of the following social media platforms most often: Instagram: n = 430 (87%), TikTok: n = 299 (61%), Snapchat: n = 237 (48%), YouTube: n = 166 (34%), WhatsApp: n = 160 (32%), Facebook: n = 106 (22%). The reported overall time spent on these platforms did not differ between genders (*p* = 0.55, d = 0.05).

### 3.3. Construct Validity

Convergent validity of FoBO was demonstrated by its positive correlation with FoMO (r = 0.58). Moreover, the relationships (r = 0.28 to r = 0.40) with markers of psychological distress (reported symptoms of depression, anxiety, somatization) are consistent with the construct’s maladaptive quality. A positive relationship (r = 0.26) with time spent on social media is also expected considering a higher exposure to the perfectly curated online lives of others. Divergent validity is shown by the negligible associations to conceptually unrelated constructs like extraversion.

## 4. Discussion

In this study, we developed the first questionnaire designed to assess the tendency to experience fear of being ordinary (FoBO). Conceptually, this construct overlaps with fear of missing out (FoMO), as both are rooted in social needs for status and stem from a perceived lack of desirable experiences. Additionally, both are associated with social media use, which often results in overexposure to idealized portrayals of others’ lives (Baker et al., 2016).

Despite these similarities, FoMO and FoBO differ in important ways. FoMO relates to everyday situations, such as staying connected or meeting friends, and is primarily driven by the need for belonging (Przybylski et al., 2013). In contrast, FoBO appears to be more existential in nature, involving concerns about one’s overall significance and impact on the world (e.g., “The thought of not having a lasting impact on the world makes me nervous”). While FoMO is motivated by the desire to be accepted, included, and connected with a peer group, FoBO centers on the pursuit of uniqueness and meaningful distinction.

This desire for significance and distinctiveness also aligns with certain narcissistic personality facets, such as grandiosity and the need for admiration. However, the correlation between scores on the new questionnaire and the Narcissistic Personality Inventory (NPI) was only modest (r = 0.21), suggesting that FoBO may not be strongly related to traditional narcissistic traits. This modest correlation could be due to the fact that FoBO is characterized more by a fear of mediocrity than by a desire for superiority (von Collani, 2008). In future studies, it may be particularly valuable to examine “vulnerable narcissism”—a subtype marked by insecurity, heightened sensitivity to criticism, and fragile self-esteem—as it may share a deeper psychological link with FoBO than grandiose narcissism does (Crowe et al., 2018).

Overall, our results on FoBO are comparable with those of previous studies on FoMO when it comes to associations with Big Five traits and psychological distress (Zhang et al., 2024; Elhai et al., 2020). The same is true for the positive association found with social media use (Rozgonjuk et al., 2020). Building on this finding, future studies should investigate whether people high in FoBO tend to gravitate towards social media and to what extent social media use may exacerbate FoBO. While we found positive associations between FoBO and indicators of psychological vulnerability, such as neuroticism, as well as somatic, depressive, and anxiety symptoms, it remains unclear whether moderate levels of FoBO might also be linked to positive real-world outcomes. In some cases, FoBO may serve as a motivational force, prompting individuals to pursue richer, more meaningful lives. However, existing research suggests that younger individuals tend to engage in more upward than downward social comparisons on social media, which can heighten dissatisfaction and self-doubt (Burnell et al., 2024). These dynamics appear to be more pronounced among females. Twenge and Martin (2020) found in their large-scale survey of adolescents (n = 221,096) that girls were more active on social media, whereas boys tended to engage more in gaming activities. Although females in the present study did not report spending more time on social media than males, they felt more influenced by it, as indicated by higher scores on the FoBO subscale “Social Media Comparisons.”

Several limitations of the present investigation should be acknowledged. First, the study relied on a predominantly young, student-adjacent convenience sample from Central Europe, which may shape both the experience and expression of FoBO. Therefore, replication in more diverse samples, including non-student populations, middle-aged and older adults, individuals from different socioeconomic backgrounds, and participants from non-European cultural contexts, is an important direction for future research. At the same time, the recruitment strategy was closely aligned with the population for which the construct was initially developed. FoBO specifically concerns social comparison processes and perceived pressures associated with contemporary social media environments. Young adults with regular social media use therefore represent a particularly relevant population in which to investigate and initially validate this construct.

Second, this was the very first use of the FoBO questionnaire and our sample size was relatively small. While our results seem promising, future studies should further test the scale’s validity.

Third, the study employed a correlational design; therefore, no causal conclusions can be drawn. The cross-sectional approach precludes determining whether greater social media use contributes to higher FoBO or, conversely, whether individuals with higher FoBO are more likely to engage in social comparison. We therefore suggest investigating temporal precedence and predictive associations with an ecological momentary assessment (EMA) design in a subsequent study. A temporal network model could then be used to examine whether higher-than-usual social media use at time t predicts higher levels of FoBO at time t + 1, while controlling for prior levels of FoBO.

Fourth, the exclusive use of self-report measures may introduce shared method variance. Future studies could therefore complement self-reported social media use with objective smartphone-based measures, such as screen-time or app-usage statistics, to obtain more accurate estimates of participants’ actual social media use. At the same time, the use of self-report instruments was appropriate for the constructs assessed in the present study, which primarily concerned participants’ subjective experiences and attitudes.

## 5. Conclusions

The brief FoBO questionnaire presented here provides an efficient way to assess the previously underexplored concept of koinophobia. The measure provides a useful tool for further research on the relationship between social media use and this specific fear, as well as for evaluating potential strategies to mitigate it.

## Figures and Tables

**Table 1:** Descriptive statistics and factor loadings from the exploratory factor analysis.

		Factor						
Item		1	2	M	SD	Skewness	Excess Kurtosis	Min–Max
1	The success of other people strongly influences my self-esteem.			2.30	1.18	0.51	−0.75	1–5 *
2	I am afraid of leading a life that goes unnoticed by others.	0.53		2.34	1.23	0.50	−0.90	1–5
3	The thought of not leaving a lasting impact on the world makes me nervous.	0.73		2.24	1.28	0.76	−0.53	1–5
4	I worry that my achievements might lose their significance over time.	0.69		2.42	1.33	0.46	−1.01	1–5
5	I am concerned that my life will have no meaning if I do not achieve fame.	0.66		1.80	1.05	1.22	0.69	1–5
6	The idea of living an ordinary life without notable success scares me.	0.82		2.52	1.38	0.39	−1.14	1–5
7	The thought of following a conventional life path fills me with concern.	0.69		2.45	1.27	0.50	−0.76	1–5
8	Social media motivates me to achieve more in my life.			2.32	1.21	0.49	−0.84	1–5
9	I feel dissatisfied when I see on social media that other people are living more exciting lives than I am.		0.83	2.27	1.28	0.62	−0.87	1–5
10	When I look at other people’s lives on social media, I feel that mine is boring.		0.88	2.32	1.32	0.54	−1.01	1–5
11	Social media have a major influence on how satisfied I am with my life.		0.71	2.15	1.19	0.73	−0.49	1–5
	Eigenvalue	2.96	2.14					
	Percentage of variance explained	32.87	23.77					

Note. Loadings were obtained using principal axis factoring with oblimin rotation. The greyed-out items were removed in prior iterations of the EFC, * rating scale: 1 = does not apply at all, 5 = applies completely; M: mean; SD: standard deviation; n = 311. For the exact item wording, see Appendix A.

**Table 2:** Internal consistencies, means, standard deviations, and Pearson correlations with the FoBO total scale and its subscales.

			Correlations with FoBO		
Measure	ω 95% CI [LB, UB]	M (SD)	FoBO (Total Score)	Fear of Insignificance	Social Media Comparisons
FoBO_total	0.86 [0.85, 0.88]	2.32 (0.91)			
FoBO_insignificance	0.87 [0.84, 0.89]	2.33 (1.02)	0.93 **		
FoBO_social_media	0.84 [0.81, 0.87]	2.31 (1.13)	0.75 **	0.44 **	
Narcissism	0.84 [0.82, 0.87]	2.95 (0.81)	0.21 **	0.29 **	−0.02
Fear of missing out	0.83 [0.80, 0.85]	2.68 (0.80)	0.58 **	0.47 **	0.55 **
Extraversion		3.16 (0.97)	0.01	0.04	−0.04
Neuroticism		3.19 (1.11)	0.34 **	0.24 **	0.39 **
Conscientiousness		3.30 (0.95)	−0.19 **	−0.16 **	−0.18 **
Openness to experience		3.71 (0.98)	−0.02	0.03	−0.09 *
Agreeableness		3.22 (0.83)	−0.12 *	−0.13 **	−0.05
Somatization	0.78 [0.74, 0.81]	3.73 (3.83)	0.28 **	0.27 **	0.20 **
Depression	0.87 [0.85, 0.89]	5.48 (5.09)	0.40 **	0.34 **	0.36 **
Anxiety	0.81 [0.78, 0.84]	5.26 (4.23)	0.32 **	0.30 **	0.24 **
Age (years)		21.93 (8.46)	−0.26 **	−0.25 **	−0.18 **
Social media use (hours/day)		3.39 (2.05)	0.26 **	0.19 **	0.27 **

Note. FoBO: fear of being ordinary; CI: confidence interval; LB: lower bound; UB: upper bound. The 95% confidence intervals for McDonald’s omega were estimated using 1000 bootstrap samples; ω: McDonald’s omega; M (SD): mean (standard deviation); n = 491; asterisks indicate significance levels, *p* < 0.05 *, *p* < 0.001 **.

## Data Availability

Data are provided by the corresponding author (anne.schienle@uni-graz.at) upon request.

## Supplementary Materials

The following supporting information can be downloaded at: https://www.mdpi.com/article/10.3390/ejihpe16100166/s1, Table S1. Descriptive statistics and factor loadings from the confirmatory factor analysis; Table S2. Means, standard deviations, skewness, kurtosis, and minimum–maximum values; Table S3. Bivariate correlations of all included measures.

## Appendix A. Fear of Being Ordinary Questionnaire (FoBOQ)—Translation

Please indicate how much the following statements apply to you.

1. I am afraid of leading a life that goes unnoticed by others.
2. The thought of not leaving a lasting impact on the world makes me nervous.
3. I worry that my achievements might lose their significance over time.
4. I am concerned that my life will have no meaning if I do not achieve fame.
5. The idea of living an ordinary life without notable success scares me.
6. The thought of following a conventional life path fills me with concern.
7. I feel dissatisfied when I see on social media that other people are living more exciting lives than I am.
8. When I look at other people’s lives on social media, I feel that mine is boring.
9. Social media have a major influence on how satisfied I am with my life.

Response format: 1–5 (with endpoint labeling 1 = does not apply at all; 5 = applies completely). Scoring procedure: compute mean scores (subscale insignificance; mean (items, 1, 2, 3, 4, 5, 6); comparisons; mean (items 7, 8, 9).
