# Supplementary material for: Fear of Being Ordinary: Development and Validation of a Questionnaire
Source: Eur J Investig Health Psychol Educ. 2026 Oct 19;16(10):166. doi: 10.3390/ejihpe16100166 (PMC13703257; doi:10.3390/ejihpe16100166)
Supplement: Supplementary file 1 [file ejihpe-16-00166-s001.zip › ejihpe-4560986-supplementary.pdf]

European Journal of
Investigation in Health, Psychology and Education

Supplementary Material

Supplementary Table S1 Descriptive statistics and factor loadings from the confirmatory factor analysis.

Item		Standardized factor loading β 95% CI [LB, UB]	Unstandardized factor loading 95% CI [LB, UB]	M	SD	Skewness	Excess kurtosis	Min-Max
Fear of Insignificance (endpoint labeling: 1 = does not apply at all; 5 = applies completely)								
2	I am afraid of leading a life that goes unnoticed by others.	0.75 [0.69, 0.81]	1.00 [1.00,1.00]	2.25	1.30	0.70	-0.71	1-5
3	The thought of not leaving a lasting impact on the world makes me nervous.	0.74 [0.68, 0.80]	0.97 [0.82, 1.12]	2.12	1.29	0.87	-0.47	1-5
4	I worry that my achievements might lose their significance over time.	0.69 [0.63, 0.76]	0.94 [0.78, 1.09]	2.37	1.32	0.58	-0.91	1-5
5	I am concerned that my life will have no meaning if I do not achieve fame.	0.78 [0.73, 0.83]	0.93 [0.80, 1.07]	1.89	1.17	1.18	0.33	1-5
6	The idea of living an ordinary life without notable success scares me.	0.77 [0.71, 0.83]	1.05 [0.89,1.21]	2.50	1.33	0.36	-1.15	1-5
7	The thought of following a conventional life path fills me with concern.	0.62 [0.54, 0.69]	0.85 [0.69, 1.01]	2.56	1.35	0.39	-1.02	1-5
Social Media Comparisons (endpoint labeling: 1 = does not apply at all; 5 = applies completely)								
9	I feel dissatisfied when I see on social media that other people are living more exciting lives than I am.	0.87 [0.82, 0.92]	1.00 [1.00, 1.00]	2.45	1.30	0.39	-1.07	1-5
10	When I look at other people's lives on social media, I feel that mine is boring.	0.82 [0.76, 0.87]	0.98 [0.85, 1.12]	2.51	1.36	0.34	-1.19	1-5
11	Social media have a major influence on how satisfied I am with my	0.70 [0.64, 0.77]	0.75 [0.63, 0.87]	2.11	1.21	0.87	-0.28	1-5

	life.							

Note. CI: confidence interval; LB: lower bound; UB: upper bound; M: mean; SD: standard deviation; n = 311.

Supplementary Table S2. Means, standard deviations, skewness, kurtosis, and minimum–maximum values

Measure	M (SD)	Skewness	Excess kurtosis	Min-Max
FoBO (total score)	2.32 (0.91)	0.37	-0.68	1-5
Fear of Insignificance	2.33 (1.02)	0.49	-0.56	1-5
Social Media Comparisons	2.31 (1.13)	0.52	-0.81	1-5
Narcissism	2.95 (0.81)	0.02	-0.39	1-5
Fear of missing out	2.68 (0.80)	0.27	-0.33	1-5
Extraversion	3.16 (0.97)	-0.03	-0.64	1-5
Neuroticism	3.19 (1.11)	-0.17	-0.87	1-5
Conscientiousness	3.30 (0.95)	-0.21	-0.54	1-5
Openness to experience	3.71 (0.98)	-0.44	-0.60	1-5
Agreeableness	3.22 (0.83)	-0.16	-0.23	1-5
Somatization	3.73 (3.83)	1.46	2.18	0-20
Depression	5.48 (5.09)	1.20	0.97	0-24
Anxiety	5.26 (4.23)	1.12	1.19	0-23
Age (years)	21.93 (8.46)	1.98	4.67	14-65
Social media use (hours/day)	3.39 (2.05)	1.61	6.18	0-16

Note. FoBO: Fear of Being Ordinary; Fear of Insignificance and Social Media Comparisons refer to the two FoBO subscales; M (SD): mean (standard deviation); n = 491.

https://doi.org/10.3390/ejihpe16100166

European Journal of Investigation in Health, Psychology and Education

Supplementary Table S3 Bivariate correlations of all included measures

	1	2	3	4	5	6	7	8	9	10	11	12	13	14	15
1 FoBO (total score)	-														
2 Fear of Insignificance	0.93***	-													
3 Social Media Comparisons	0.75***	0.44***	-												
4 Narcissism	0.21***	0.29***	-0.02	-											
5 Fear of missing out	0.58***	0.47***	0.55***	0.09	-										
6 Extraversion	0.01	0.04	-0.04	0.34***	0.15**	-									
7 Neuroticism	0.34***	0.24***	0.39***	-0.27***	0.32***	-0.17***	-								
8 Conscientiousness	-0.19***	-0.16***	-0.18***	0.05	-0.17***	0.12**	-0.08	-							
9 Openness to experience	-0.02	0.03	-0.09*	0.11*	0.00	0.08	-0.01	0.03	-						
10 Agreeableness	-0.12*	-0.13**	-0.05	-0.14**	-0.02	0.08	-0.05	0.03	0.06	-					
11 Somatization	0.28***	0.27***	0.20***	0.02	0.27***	-0.03	0.35***	-0.13**	0.07	-0.14**	-				
12 Depression	0.40***	0.34***	0.36***	-0.15***	0.33***	-0.16***	0.44***	-0.21***	0.01	-0.14**	0.53***	-			
13 Anxiety	0.32***	0.30***	0.24***	-0.08	0.29***	-0.05	0.52***	-0.11*	0.10*	-0.13**	0.67***	0.66***	-		
14 Age (years)	-0.26***	-0.25***	-0.18***	-0.20***	-0.19***	-0.07	-0.13**	0.13**	0.01	0.05	-0.23***	-0.16***	-0.17***	-	
15 Social media use (hours/day)	0.26***	0.19***	0.27***	0.14**	0.26***	0.07	0.13**	-0.22***	-0.10*	0.03	0.16***	0.20***	0.13**	-0.28***	-

Note. FoBO: Fear of Being Ordinary; Fear of Insignificance and Social Media Comparisons refer to the two FoBO subscales; n = 491; asterisks indicate significance levels; * $p < 0.05$, ** $p < 0.01$, *** $p < 0.001$.
